# Smart Wearables for the Detection of Cardiovascular Diseases: A Systematic Literature Review

**DOI:** 10.3390/s23020828

**Published:** 2023-01-11

**Authors:** Mohammad Moshawrab, Mehdi Adda, Abdenour Bouzouane, Hussein Ibrahim, Ali Raad

**Affiliations:** 1 Département de Mathématiques, Informatique et Génie, Université du Québec à Rimouski, 300 Allée des Ursulines, Rimouski, QC G5L 3A1, Canada; 2 Département d’Informatique et de Mathématique, Université du Québec à Chicoutimi, 555 Boulevard de l’Université, Chicoutimi, QC G7H 2B1, Canada; 3 Institut Technologique de Maintenance Industrielle, 175 Rue de la Vérendrye, Sept-Îles, QC G4R 5B7, Canada; 4 Faculty of Arts & Sciences, Islamic University of Lebanon, Wardaniyeh P.O. Box 30014, Lebanon

**Keywords:** cardiovascular diseases, smart wearables, sensors, body sensor networks, machine learning, smart health, wide body area networks

## Abstract

**Background:** The advancement of information and communication technologies and the growing power of artificial intelligence are successfully transforming a number of concepts that are important to our daily lives. Many sectors, including education, healthcare, industry, and others, are benefiting greatly from the use of such resources. The healthcare sector, for example, was an early adopter of smart wearables, which primarily serve as diagnostic tools. In this context, smart wearables have demonstrated their effectiveness in detecting and predicting cardiovascular diseases (CVDs), the leading cause of death worldwide. **Objective:** In this study, a systematic literature review of smart wearable applications for cardiovascular disease detection and prediction is presented. After conducting the required search, the documents that met the criteria were analyzed to extract key criteria such as the publication year, vital signs recorded, diseases studied, hardware used, smart models used, datasets used, and performance metrics. **Methods:** This study followed the PRISMA guidelines by searching IEEE, PubMed, and Scopus for publications published between 2010 and 2022. Once records were located, they were reviewed to determine which ones should be included in the analysis. Finally, the analysis was completed, and the relevant data were included in the review along with the relevant articles. **Results:** As a result of the comprehensive search procedures, 87 papers were deemed relevant for further review. In addition, the results are discussed to evaluate the development and use of smart wearable devices for cardiovascular disease management, and the results demonstrate the high efficiency of such wearable devices. **Conclusions:** The results clearly show that interest in this topic has increased. Although the results show that smart wearables are quite accurate in detecting, predicting, and even treating cardiovascular disease, further research is needed to improve their use.

## 1. Introduction

Healthcare has always been one of the most important issues that people have cared about. Given the prevalence of diseases and their impact on people’s lives, researchers are always looking for methods to improve medical services and promote public health. In addition, the aging population, shortage of medically trained personnel, lack of equity in services, epidemic planning, and a host of other problems hinder the growth of public health worldwide [1]. However, advances in information and communication technology (ICT) offer effective answers to these challenges. In this context, artificial intelligence (AI) is considered the most promising tool for improving healthcare, as it has the potential to be used in virtually all areas of medicine [2] and will transform healthcare for patients and communities [3]. This enormous contribution is not due to magic, but to AI’s data-processing capabilities, which surpass those of humans, especially when large computations are performed in a short period of time. Even though the majority of AI applications in healthcare were developed after 2008 [4], their importance is obvious. First, AI has improved the learning capabilities of computers and humans, leading to improved diagnostic and healthcare procedures [5]. In addition, AI technologies are able to accept common sense, extract information from raw data, use human-like thought processes, deal with inaccuracies, adapt to a rapidly changing environment, and even act on their knowledge [2]. These characteristics enable AI tools to think and behave similar to humans at a virtually unparalleled level, allowing them to articulate clinical patterns and visions beyond human capabilities [3]. Combining AI capabilities with human intelligence, sometimes referred to as augmented intelligence, is probably the most effective way to improve healthcare services [3].

### 1.1. Cardiovascular Diseases Latest Figures

**Figure 1:**
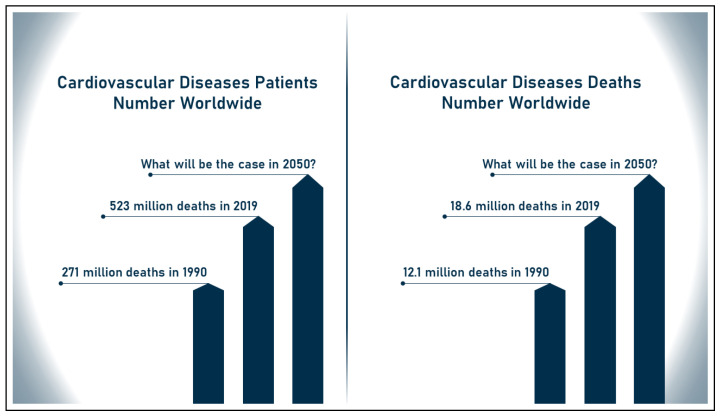
Increase in number of patients and deaths due to CVDs.

Cardiovascular diseases (CVDs) are the leading cause of death and are hence recognized as the most dangerous disease in the world. According to the most recent World Health Organization (WHO) statistics on heart disease, the number of CVD patients worldwide has increased from 271 million to 523 million between 1990 and 2019, and the number of deaths caused by this disease has increased from 12.1 million to 18.6 million during the same period, accounting for 32% of global mortality in 2019 [6]. For example, in the United States, a person dies from heart disease at least every 34 s [7], and in Canada, a person dies at least every 5 min [8]. Moreover, cardiovascular disease is a major cause of both health conflict and economic suffering. According to the Medical Expenditure Panel Survey, the total cost of CVDs in the United States between 2017 and 2018 was estimated at USD 378.0 billion, including USD 226.0 billion in expenditures and USD 151.8 billion in lost future productivity [9]. Figure 1 illustrates the increase in the number of patients and deaths due to cardiovascular disease worldwide between 1990 and 2019.

### 1.2. CVDs Detection: From Classic to Technology-Assisted

Due to their potentially fatal nature, cardiovascular diseases need the development of efficient solutions that allow early diagnosis and, ideally, prediction of their onset. The predictive power of modern technologies could help reduce the global prevalence of CVDs. Traditional methods for diagnosing these diseases include electrocardiogram, echocardiography, coronary angiography, stress testing, magnetic resonance imaging, or intracoronary ultrasonography. However, new technologies are improving health services and facilitating the detection of cardiovascular disease, particularly information and communication technologies (ICTs) and the development of artificial intelligence (AI) and its derivatives. The novel approaches of AI in cardiology have proven to be successful in providing fast, accurate, and less erroneous patient care, which has significant medical and financial implications. It is more effective and widely used, as the tools and applications offered are at the level of an expert using real-world data. In general, AI has fantastic potential to transform cardiology in the near future and is often seen as the next revolutionary step in the field due to its potential to accelerate and improve patient care. Moreover, AI will soon revolutionize cardiovascular health, as its tools have the potential to outperform experts in detecting and predicting cardiovascular disease [10,11,12]. Therefore, smart wearables that combine AI and ICT are expected to be very useful in cardiovascular disease detection and prediction.

### 1.3. Smart Wearables: Definitions and Overview

Smart wearables, also known as smart wearable technology or wearable gadgets, are a new breed of compact, rugged, and efficient computing devices made possible by the rapid growth of information and communication technologies and the advancement of electronics, particularly microprocessors. These devices are being hailed as the next generation of ubiquitous technology after smartphones, as they allow access to data at any time and from any location. The topic of smart wearables has evolved rapidly in recent years, and their technologies are now applicable in many other fields [13,14,15,16]. This section provides a definition of “smart wearables” and a brief overview of the history of wearable technology. In addition, various categories of smart wearables are discussed in the upcoming sections.

#### 1.3.1. Smart Wearables: Brief History

In 1950, Alan Turing asked the now famous question “Can machines think?” which marked the beginning of the era of “Smart Machines” [17]. Since then, researchers around the world have attempted to answer this question by turning computers into intelligent devices. Despite its widespread use, the term “Smart” is not uniformly defined and is presented in different ways by different scholars [18]. In [19], “Smart” devices are defined as embedded sensors, processors, and network devices that give smart things the ability to behave based on their own knowledge. In addition, Ref. [20] defines them as objects that can learn from their environment and interact with humans. Different definitions focus on the capabilities of the devices. For example, smart wearables are defined by the authors in [21,22] as devices that can be worn by the user at all times to monitor factors such as personal data, vital signs, locations, environment, movements, and more. In this context, a shoe-sized computer developed by Edward Thorp and Claude Shannon in 1961 is widely considered to be the first ever wearable computing device [23,24]. In the 1980s, Steve Mann developed EyeTap glasses that displayed computer-generated images in one eye and added textual information to the user’s visual experience [25]. Subsequently, in 1996, the U.S. Department of Defense Navy funded a study to monitor the vital signs of its troops [26,27], which is widely considered a defining moment in the history of smart wearables. Since then, smart wearables have gradually evolved from invasive, heavy, and huge technologies to more adaptable, compact, and lightweight devices. This is because researchers have expanded their projects in this field to different areas of life such as health, fitness, sports, fashion, and even other sectors.

#### 1.3.2. Classification of Smart Wearables

Over the past few decades, there have been more than a thousand studies on smart wearables. However, smart wearables cannot be classified into a specific category. Accordingly, smart wearables are divided into six groups, as described by the authors of Ref. [28]:

- Medical;
- Industrial;
- Lifestyle;
- Fitness;
- Entertainment;
- Gaming.

On the other hand, the smart wearables were categorized by the authors of Ref. [29] according to their personal features rather than their function. They provided examples of the three categories into which they fall:

- Watch-type;
- Necklace or wristband-type;
- Headmount display-type.

**Figure 2:**
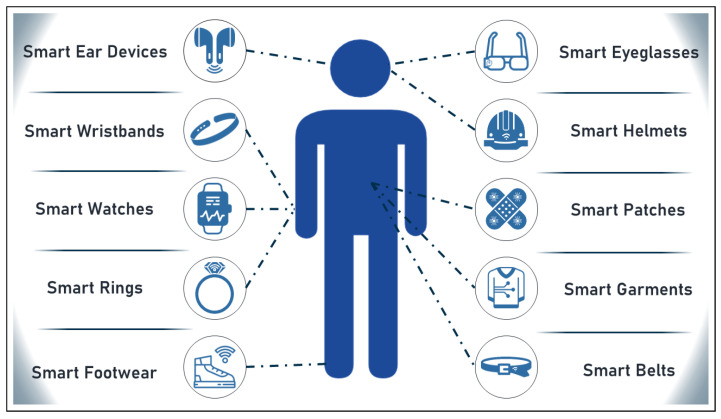
Set of commonly known wearables.

However, other technologies, such as electronic patches and health apparel, could not fit within this classification. Therefore, a set of commonly known wearables are displayed in Figure 2 below.

### 1.4. Role of Smart Wearables in CVDs

Over the past decade, smart wearables have been increasingly used as health solutions. Their effectiveness and proliferation has been fueled by advances in performance, size, style, and durability, among other factors. Examples of smart wearables used to diagnose, track, and treat cardiovascular disease include wristbands, patches, headbands, eyeglasses, and necklaces. The implications of CVD wearables are many. For example, they enable continuous and long-term recording of functional or physiological data, leading to more accurate diagnosis and better health outcomes for patients. In addition, they enable the collection of necessary data in locations other than physicians’ offices or hospitals, expanding the capacity of healthcare facilities to serve larger numbers of patients over longer periods of time. More importantly, the continuous monitoring capabilities of smart wearables enable more sophisticated knowledge of an individual patient’s physiological state and ongoing activity, paving the way for more personalized healthcare and treatment. The devices also became less bulky and aesthetically pleasing, making them less intrusive and more suitable as everyday wearables. One way that smart wearables such as smartphones are benefiting from the widespread use of other devices is through pairing [30,31,32,33]. Some reasons for the success of smart wearables adoption and their success points are listed in Table 1 below.

### 1.5. Outline and Main Contributions of This Article

In this article, the use of smart wearables in the detection, prediction, and treatment of cardiovascular disease was investigated. For this purpose, a systematic literature review was conducted, following the methodology that is explained in Section 2. Subsequently, in Section 3, the results of the performed search are presented. Later, the obtained results are analyzed in Section 4 and discussed in Section 5. Then, the challenges hindering the progress in the use of smart wearables are discussed, and future perspectives to solve these challenges are presented. Finally, the article is concluded with a concluding section. To the best of our knowledge, there are no systematic reviews addressing the potential of smart wearables for early diagnosis of CVDs. For example, in [34], the authors investigated the application of AI in smart wearables for cardiovascular disease detection. However, the focus of their research was on smart models rather than hardware; the obstacles that have slowed the development of this field are barely addressed, and the same is true for future prospects. Furthermore, in [35,36], the authors explored the use of smart wearables in life course research, but they did not systematically explore the field or provide a complete vision of contextual implementations. Motivated by the large role that smart wearables play in various aspects of daily life and by the lack of a systematic literature review discussing their role in predicting cardiovascular disease, this article therefore attempts to answer the following questions:

- What are the applications of using smart wearables to detect and predict cardiovascular disease?
- What are the different aspects such as hardware and software used in these implementations?
- To what extent are these implementations feasible?
- What are the challenges and limitations in this area?
- What future perspectives can be pursued to improve the use of smart wearables in CVDs management?

Therefore, this article answers the above questions and thus contributes to academic knowledge by:

- Systematically reviewing the use of smart wearables in the treatment of cardiovascular disease;
- Analyzing and discussing the reviewed implementations in a way that facilitates the identification of opportunities for improvement in this area;
- Naming the barriers to progress in this area;
- Proposing solutions that can be used to address these barriers;
- Presenting a collection of research questions and findings that could serve as a starting point for future research.

## 2. Research Methodology

This section details and explains the methodology used to conduct the systematic review. The steps described here can be used to conduct the same search and review the results or repeat the search in a different time period.

### 2.1. Eligibility Criteria

In conducting this review, PRISMA (Preferred Reporting Items for Systematic Reviews and Meta-Analyses ) [37] was used as a guide for preparing a systematic literature review. The structure of this review was based on the latest PRISMA checklist (PRISMA Checklist 2020) [38]. In accordance with PRISMA standards, multiple sources were searched for papers that met the scope of this review. Four variables were used to select these materials. To be considered reputable, a paper must address artificial intelligence or related fields, present a smart wearable solution, address healthcare, and focus on cardiovascular diseases. In addition, the material should have been published in a peer-reviewed journal or as a conference proceedings. In addition, only documents published between January 2010 and October 2022 were considered. As a final eligibility criterion, an English language filter was applied to eligible papers.

### 2.2. Information Sources

Several academic abstract and citation databases for peer-reviewed literature were used, including IEEE, PubMed, and Scopus Elsevier, to ensure superior results and to cover the largest number of documents possible. Each of these three databases provides access to millions of documents and has powerful, sophisticated search tools to facilitate thorough literature searches.

### 2.3. Search Strategy

In order to conduct a thorough search of the above materials, three queries were formulated. While these queries all follow the same logical structure, they use different syntaxes to comply with the different rules imposed by each data source. Targeted articles are found at the intersection of four query blocks, each defining a different topic of interest. AI, health, wearables, and CVDs (or related areas) are the four basic focus areas. The phrase “AND” was used to combine areas for a more effective query, while the term “OR” was used to combine different terms within each area. The three queries used to find what is being searched for are as follows:

- IEEE: ((("ARTIFICIAL INTELLIGENCE" OR "SMART AGENTS" OR "SMART MACHINES" OR "INTELLIGENT" OR "DEEP LEARNING" OR "MACHINE LEARNING" OR "NEURAL NETWORK") AND ("HEALTH*" OR "DISEASE" OR "ILL*" OR"CARE") AND ("WIRELESS SENSORS NETWORK" OR "SMART SENSORS" OR "BODY AREA NETWORK" OR "WEARABLE" OR "SENSOR") AND ("CARDIOLOGY" OR "CARDIOVASCULAR" OR "HEART" OR "CARDI*"))).
- PubMed: ((ARTIFICIAL INTELLIGENCE) OR (SMART AGENTS) OR (SMART MACHINES) OR (INTELLIGENT) OR(DEEP LEARNING) OR (MACHINE LEARNING) OR (NEURAL NETWORK)) AND ((HEALTH) OR (DISEASE) OR (ILL) OR(CARE) OR (HEALTHCARE)) AND ((WIRELESS SENSORS NETWORK) OR (SMART SENSORS) OR(BODY AREA NETWORK) OR (WEARABLE) OR (SENSOR)) AND ((CARDIOLOGY) OR (CARDIOVASCULAR) OR (HEART) OR(CARDIAC)).
- Scopus: TITLE-ABS-KEY(((artificial intelligence) OR (smart agents) OR (smart machines) OR (intelligent) OR (deep learning) OR (machine learning) OR (neural network)) AND ((health*) OR (disease) OR (ill*) OR (care)) AND ((wireless sensors network) OR (smart sensors) OR (body area network) OR (wearable) OR (sensor)) AND ((cardiology) OR (cardiovascular) OR (heart) OR (cardi*))) AND (LIMIT-TO (SRCTYPE, “j”) OR LIMIT-TO (SRCTYPE, “p")) AND (LIMIT-TO(DOCTYPE, “cp”) OR LIMIT-TO(DOCTYPE, “ar”)) AND (LIMIT-TO( LANGUAGE, “English”)) AND (LIMIT-TO (PUBYEAR, 2021) OR LIMIT-TO (PUBYEAR, 2020) OR LIMIT-TO (PUBYEAR, 2019) OR LIMIT-TO (PUBYEAR, 2018) OR LIMIT-TO (PUBYEAR, 2017) OR LIMIT-TO (PUBYEAR, 2016) OR LIMIT-TO (PUBYEAR, 2015) OR LIMIT-TO (PUBYEAR, 2014) OR LIMIT-TO (PUBYEAR, 2013) OR LIMIT-TO (PUBYEAR, 2012) OR LIMIT-TO (PUBYEAR, 2011) OR LIMIT-TO (PUBYEAR, 2010)).

In the case of Scopus, the query was used as described above to retrieve the results. However, in IEEE and PubMed, additional filters were applied through the graphical user interface. In both sources, a “Year” filter was added to limit the selection to articles between 2010 and 2021. However, in IEEE, articles with the types “Conferences” and “Journals” were selected, whereas in PubMed, articles with the types “Clinical Trial” and “Journal Article” were selected. Finally, a filter was performed in the PubMed interface to limit the documents to those published in English. On the other hand, Scopus offers the possibility to limit the search to the title, abstract, or keywords, while the other two sources perform the search in the whole text of the document. It is worth noting that the search was performed in October 2021.

### 2.4. Selection Process

The data extracted from the records were selected in three steps to determine which files were relevant to this analysis. The first step was to review the titles and abstracts of all documents to determine if they were relevant to the topic of this study. The documents that passed this step were then downloaded. In a second step, we reviewed the downloaded files to quickly verify their content and determine if they were relevant to our evaluation. The documents selected in this phase are the ones that are examined in detail. Finally, the documents were researched and evaluated to extract the data needed to demonstrate the development of smart wearables for CVDs.

## 3. Results

The steps mentioned in the previous section led to a systematic result. The results of this search are listed in this section.

### 3.1. Study Selection

**Figure 3:**
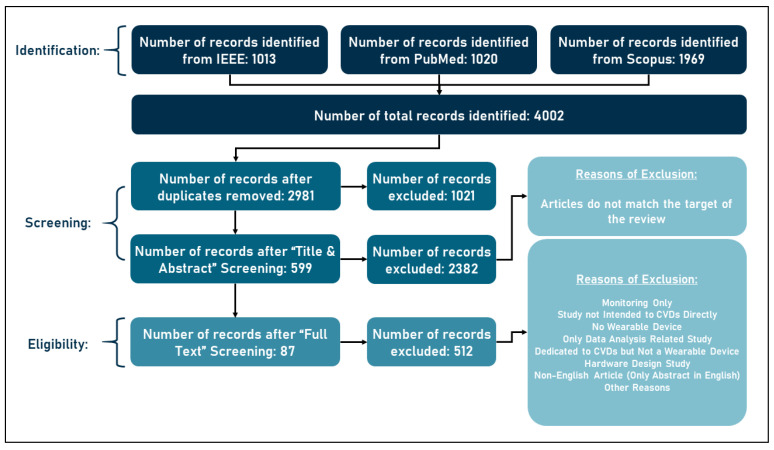
Flow of information through the different phases of a systematic review.

Initially, 4002 documents were identified from the three libraries based on the above searches. The search on IEEE yielded 1013 documents, on PubMed 1020, and on Scopus 1969, after which duplicate entries were excluded, removing 1021 and leaving 2981 documents. Then, the aforementioned selection procedure was applied, excluding 2382 documents on the basis of irrelevance and advancing 599 to the next stage. Documents were classified as irrelevant if they met the search criteria or if they contained the search terms specified in the search queries but did not deal with cardiovascular disease or were not wearable systems. In the second phase, full-text screening, the 599 documents were downloaded and skimmed to assess their suitability. In this phase, 512 documents were excluded for various reasons, and 87 documents were deemed suitable for this review. All these details are shown in Figure 3 below that matches the PRISMA diagram (information flow through the different phases of a systematic review) [37].

### 3.2. Study Characteristics

Following the search described above, the 87 papers deemed appropriate were carefully reviewed and examined to extract all relevant information. From each paper, the year of publication, disease(s) treated, vital signs recorded, hardware of the wearable device(s), embedded intelligent models, dataset(s) used, and outcome metrics were extracted. Table 2 below shows all retrieved details from the eligible studies, thus forming one of the main outcomes of this research, listing all implementations of smart wearables in cardiovascular disease management between 2010 and 2022 along with their relevant details.

### 3.3. Results of Individual Studies

The systems presented in the eligible studies share common features that allow for easy classification. Contextually, the studies can be divided into three categories, for example, according to whether the measuring devices used are commercially available or not. Systems in the first group use components that are not commonly available; these components were custom-made by the researchers for the study. Systems that use readily available technology and commercially available devices comprise the second category. The final category includes studies that used unspecified devices, making it impossible to determine whether or not they are now available for purchase. The following categories of systems were formed according to the devices used.

#### 3.3.1. Studies Using Custom-Built Devices

Throughout the analysis of the eligible documents, it was shown that 55 studies built their own devices using various vital sign sensors, power resources, storage resources, communications, and other technical components. Within this group, two subgroups stood out, the first of which did not name all the components used, particularly the sensor devices, but, rather, stated that they composed their own wearables from sensor devices. Thus, in [39,41,42,43,46,48,50,51,54,56,58,59,60,61,62,66,86,88,92,116,118,123], the authors proposed custom-built wearables with unspecified components. These studies were able to detect various cardiovascular diseases such as atrial fibrillation, atrial flutter, atrial premature contraction, atrial tachycardia, cardiac asystole, cardiovascular risk, fusion beats, heart attack, heart failure, hypertension, myocardial infarction, myocardial ischemia, premature atrial contractions, and premature ventricular contractions. Vital signs obtained for this purpose were radial artery audio signal, blood oxygen level, blood pressure, blood sugar level, body temperature, diastolic pressure, electrocardiogram, electroencephalogram, heart rate, motion data, oxygen saturation level, photoplethysmogram, respiratory rate, and skin temperature. In addition, the databases used for training and the performance metrics are detailed in Table 2.

On the other hand, several studies used commercially available sensors to develop their wearable devices. In this context, various sensors such as ECG, accelerometer, and other sensors were used. For example, in [53,73,104,117,125], the ECG sensors ADAS1001, Shimmer 3, BMD101, ADXL355, and ADS1291 were used in combination with other materials to build a wearable device that collects records used to detect or predict cardiovascular disease. In contrast, the authors in [114] used the DS18B20 temperature sensor and ADXL1335 accelerometer to develop the desired wearable system. In addition, the authors in [52,57,65,67,69,71,72,74,75,77,79,81,89,94,100,101,113,122] used the AD8232 ECG sensor to collect vital signs data. In these studies, as discussed in Table 2, different processing units, connector modules, and power sources were used to build the wearable device. Alternatively, in [82,84,95,97], the authors combined different sensor materials with the AD8232 ECG sensor in their wearable device. Specifically, the authors in [82,95] used the MAX30100 blood oxygen sensor in addition to the ECG sensor, whereas the authors in [84] used the ADXL345 triaxial accelerometer, and the authors in [97] used the MAX30102 pulse oximeter sensor. Other studies also used different ECG sensors, with the authors in [87] building their portable devices using the “Ternary Second-Order Delta Modulator Circuits” to acquire ECG data. In the same context, the authors in [111,124] used the sensor ECG AFE and other tools to build a wearable device capable of acquiring the necessary vital signs data. Finally, the authors of [109,119] used MAX30102 photoplethysmography and BH1790GLC optical heart rate sensors in their wearable devices, respectively.

#### 3.3.2. Studies Using Commercially Available Wearable Devices

The other group of studies consists of studies that used commercially available devices. These devices were capable of recording various vital signs such as activity parameters, blood oxygen level, blood pressure, blood glucose level, cholesterol level, electrocardiogram, electroencephalogram, electromyogram, heart rate, oxygen saturation level, photoplethysmogram, pulse plethysmogram, and respiratory rate. Depending on the type of device used, three categories can be distinguished, namely, wristband devices, belts, and others. For example, in [45,47,55,68,70,80,85,98,103,107,108,112], the authors used smartwatches and smart wristbands to record vital signs. In addition, the authors in [40,44,49,63,64,76,78,83,91,93,96,102,110,120,121] used various wearable ECG devices such as smart vests and patches. In addition, the authors in [90,99,105,106] used smart belts to collect ECG data. Overall, the devices used in all the studies mentioned in this section can be summarized in the following list:

- Alive ECG Heart Monitor;
- Amazfit Health band 1S;
- Apple Smart Watch;
- Bio Clothing One, XYZ life BC1;
- BioHarness 3.0 by Zephyr;
- ECG247 Smart Heart Sensor;
- Firstbeat Bodyguard Chest Patch 2 by Firstbeat Technologies;
- GENEActiv and Activinsights Band by Activinsights Ltd.;
- Glucose Monitor by Medtonic;
- HealthyPiV3 biosensors;
- Heart Rate sensor by Sunrom Electronics;
- IREALCARE2.0 Wearable ECG Sensor;
- Kimbolton, UK;
- Medical-Grade Wearable Embedded System Beijing Sensecho Science & Tech.;
- Wearable device provided by Medicaltech SRL;
- Moto 360;
- NanoPi Neo Plus2;
- Polar H10;
- PTN-104 PPG Sensor;
- Raspberry Pi Zero;
- Rejiva ECG Wearable Sensor;
- Rozinn RZ153+ ECG Monitor;
- Samsung Galaxy Active 2 Smart Watch;
- Samsung Galaxy Active Smart Watch;
- Samsung Gear Wearable Device;
- Samsung Simband 2 Wrist Band Smart Watch;
- Samsung Simband Wrist Band Smart Watch;
- Shimmer ECG Monitor;
- Single-Lead Heart Belt by Suunto Movesense, Suunto, Vantaa, Finland;
- Wrist-Type Pulse Wave Monitor by: Shanghai Asia & Pacific Computer Info. System.

#### 3.3.3. Studies That Did Not Specify the Devices Used

Finally, in a single study, the device used was not specified. The authors in [115] mentioned only that they used a wide body area network (WBAN) to record respiratory rate and blood oxygen levels to detect the presence of cardiovascular risk. Their study achieved 96% accuracy in the classification algorithm, demonstrating high feasibility in detecting CVDs. Unlike some of the studies excluded in the screening phases (see Section 2.4), this study mentioned that a wearable device was used, but did not specify which device was used.

## 4. Results Analysis

Studies that met the criteria for inclusion in this review, or those that specifically address the use of smart wearables for the diagnosis, prognosis, or treatment of cardiovascular disease, contain a wealth of information worthy of further investigation. In the previous section, the devices used were mentioned. However, to better understand the field, this part examines factors such as the year of publication, vital signs collected, diseases treated, smart models used, datasets used for training, etc.

### 4.1. Progress with Years

**Figure 4:**
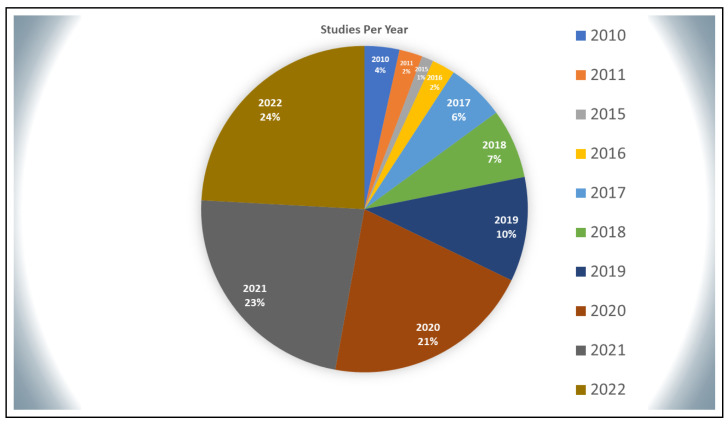
Distribution of studies per year.

Once the data are extracted from the papers, it is clear that there has been significant progress in the field of wearables for CVD research over the past four years, with 78% of the publications published in 2019 or later. During those years, a total of 68 studies were published (compared to only 19 in 2010–2018). There are nine in 2019, eighteen in 2020, twenty-one in 2021, and twenty-one in 2022. The number of publications addressing the use of smart wearables for cardiovascular disease management has jumped, reflecting both the growing interest in this area and the widespread acceptance of such devices. The data from this section are shown as a pie chart in Figure 4 below.

### 4.2. Vital Signs in Use

The electrocardiogram (ECG) is used in 69 of the systems described in the articles to diagnose disease and identify cardiac abnormalities, although many other methods have been offered. Electrocardiograms are routinely performed to check the health of the heart and quickly identify potential problems. An electrocardiogram (ECG) shows the development of the heart’s electrical activity over time. When the heart muscle cells are electrically depolarized, the heart muscle contracts. An electrocardiogram records and amplifies this electrical activity over a period of time. Studies have shown that smart watches such as the Samsung Active and Apple Watch have significant efficiency in capturing ECG signals, complementing the accuracy of ECGs performed in a doctor’s office, clinic, or hospital room. In addition, the P wave, the QRS complex, and the T wave are the three components of the ECG signal. Figure 3 shows the ECG signal in terms of these components. In a normal electrocardiogram, the heartbeat is detected by [126]:

- PR interval: measured from the beginning of the P wave to the first deflection of the QRS complex with a normal range of 120–200 ms;
- QRS complex: measured from first deflection of QRS complex to end of QRS complex at isoelectric line with a normal range of up to 120 ms;
- QT interval: measured from first deflection of QRS complex to end of T wave at isoelectric line with a normal range of up to 440 ms (though it varies with heart rate and may be slightly longer in females).

Twenty more measures, including photoplethysmogram, heart rate, and others, were also employed in addition to ECG in order to identify CVDs. Table 3 below details the frequency and utilization of these parameters across studies.

### 4.3. Diseases Targeted

**Figure 5:**
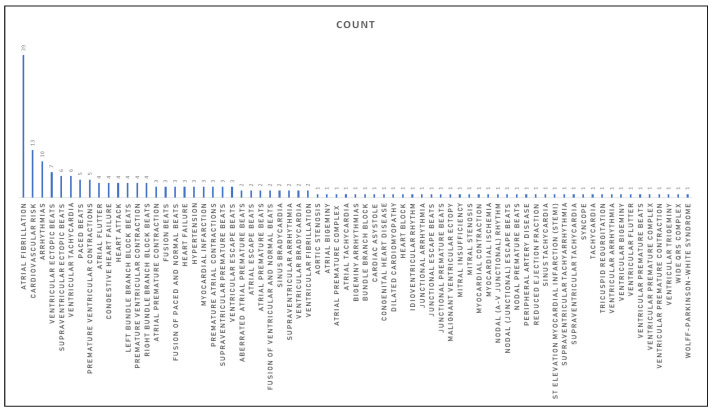
Diseases distribution per studies.

Because a single document may focus on a single disease or multiple diseases, the number of diseases studied in these publications exceeds 70. Atrial fibrillation (AFib) is the most commonly studied disease, with 39 of 87 studies addressing it. AFib is the leading cause of death and morbidity due to stroke, heart failure, thromboembolism, and reduced quality of life, and accounts for the majority of these cases [127]. Other conditions are also being studied, including premature ventricular contractions (PVCs), ventricular ectopic beats, bradycardia, paced beat (PACE), and many others. Figure 5 is a bar graph showing the number of diseases found in the 87 papers.

### 4.4. Smart Models in Use

**Figure 6:**
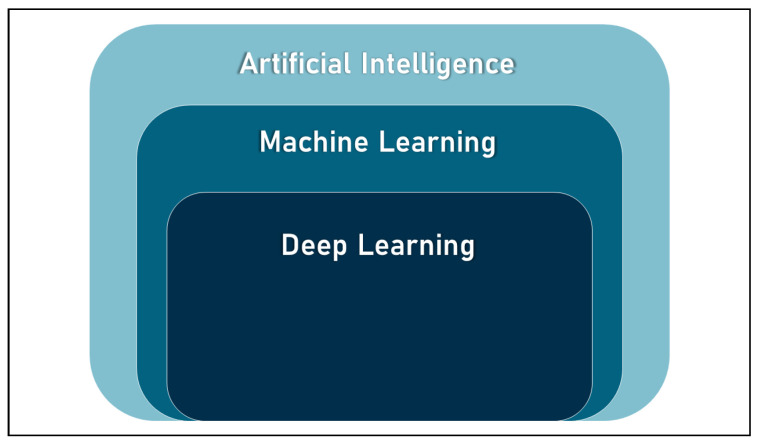
Artificial intelligence, machine learning, and deep learning relation.

It is well known that several subfields of artificial intelligence are widely used in different fields. Two of the best-known subfields of AI are machine learning (ML) and deep learning (DL); the former is described as a set of techniques that allow a machine to acquire new information and skills through learning, and the latter is a branch of machine learning that focuses on algorithms inspired by the structure and function of the brain, called artificial neural networks [128,129]. The relationship between AI, ML, and DL is illustrated in Figure 6. However, in the studies analyzed in this review, many machine learning and deep learning models were used to detect cardiovascular disease. Although each publication proposes a different method to detect the disease(s), all agree that some type of algorithm should be used to classify cardiac abnormalities. Convolutional neural networks, support vector machines, long short-term memories, and decision trees are the most commonly used algorithms.

The intelligent models of machine learning and deep learning are attracting much attention and are proving to be very practical [130,131] in the healthcare industry. With this in mind, it is of great interest to analyze the efficiency of these models in detecting CVDs. However, this task requires separate studies, as this research focuses on smart wearables as a whole system. This article aims to fill this gap by discussing the four most commonly used smart models, namely:

- Convolutional Neural Network (CNN): CNN is a kind of deep neural network used to analyze visual images. These neural networks are modeled after the neural networks of the human visual system. Neurons are the basic computational unit of a neural network, just as they are the basic functional unit of the human nervous system. In the case of convolutional neural networks, instead of normal matrix multiplication, convolution is used, a special form of mathematical operation. In addition to the input and output layers, a convolutional neural network has numerous hidden layers (a neural layer is a stack of neurons in a single row). A neuron in the input layer receives an input, analyzes it, and performs computations on it, and then transmits a nonlinear function called an activation function to produce the final output of a neuron [132];
- Support Vector Machines (SVMs): SVM is a supervised machine learning model for two-group classification problems that employs classification techniques. An SVM model is able to classify new data after receiving a set of labeled training data for each category [133];
- Long Short-Term Memory (LSTM): LSTM networks are a type of recurrent neural network (RNN) that can learn sequence dependence in sequence predictions. RNNs contain cycles that use network activations from a previous time step as inputs to influence predictions at the current time step. These activations are stored in the internal states of the network, theoretically preserving long-term contextual timing information. This method allows RNNs to use a contextual window that changes dynamically over the course of the input sequence. Complex problem domains such as machine translation, speech recognition, and others require this behavior [134];
- Decision Trees (DTs): A decision tree is a type of supervised machine learning used to make classifications or predictions based on answers to a prior set of questions. The model is a type of supervised learning, meaning that it is trained and evaluated on a dataset that contains the desired classification. Occasionally, the decision tree may not provide a definitive answer or conclusion. Instead, it may suggest possibilities from which the data scientist can make an informed choice. Because decision trees replicate human thought processes, it is often easy for data scientists to understand and explain the results [135].

The machine learning and deep learning models utilized in the studies analyzed in this review were assessed with different performance metrics such as accuracy, specificity, sensitivity, precision, recall and F1-score. These parameters are explained in detail in the literature, with the authors in [129] providing a detailed explanation in this regard, for example. These parameters can be summarized as follows [129]:

- Accuracy: the fraction of predictions that the model predicted right and is calculated by dividing the number of correct predictions by the total number of predictions.
- Specificity: is the parameter used to calculate model’s ability to predict a true negative (no cardiovascular diseases in our case) of each category available.
- Sensitivity: is the parameter used to calculate model’s ability to predict the true positives (existence of CVDs in our case) of each category available.
- Precision: is the parameter used to calculate what proportion of positive identifications (existence of CVDs in our case) was actually correct.
- Recall: is the parameter used to calculate what proportion of actual positives (existence of CVDs in our case) was identified correctly.

The performance of models used by each study is detailed in Table 2. Furthermore, the list of smart models used in smart wearables for the detection of cardiovascular diseases is mentioned in Table 4 below, along with the count of use of each model. In this context, and for more details on the potential of machine learning and deep learning models in predicting CVDs, readers are advised to refer the work of Solam Lee and his colleagues [34], which targets these models and discusses their feasibility in this domain.

### 4.5. Datasets in Use

**Figure 7:**
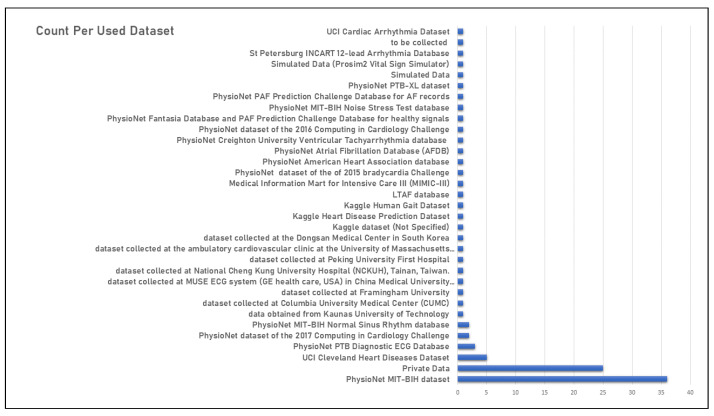
Training datasets in use.

In all 87 publications examined, at least one dataset was used to train the AI model, and this is consistently the case. In addition, it was noted that certain sources advocate the use of multiple datasets in the development and evaluation of a model. The PhysioNet MIT-BIH dataset, accessible through the PhysioNet library of publicly available medical research data, is the most popular. Of the 87 total studies, 36 used it. Another 25 studies also used researchers’ private data. Figure 7 below shows a graphical statistical representation of the frequency of use of datasets. The MIT-BIH Arrhythmia database is the first publicly available collection of standardized test material for the evaluation of arrhythmia detectors. The BIH Arrhythmia Laboratory collected these ambulatory two-channel ECG recordings from 47 patients between 1975 and 1979 and included 48 30-minute samples [136]. The PhysioNet MIT-BIH Atrial Fibrillation Database, the PhysioNet MIT-BIH Noise Stress Test Database (NSTDB), and the PhysioNet MIT-BIH Normal Sinus Rhythm Database were also consulted.

## 5. Results Discussion

This study systematically collects and analyzes the literature on the use of smart wearables for cardiovascular disease diagnosis and prognosis. However, there is more to be said about the studies discussed so far, especially in terms of their effectiveness and conformity with the latest research areas in artificial intelligence. This topic will be elaborated and explored in this section.

### 5.1. Performance, Usability, and Feasibility

To predict CVDs, many tools have been used. The wide range of research is due to the wide range of vital signs and devices used to achieve this goal. ECG, BP, HR, and temperature were all reliable predictors of cardiovascular disease. This is evidenced by the fact that the results of several studies (described in Table 2) showing the use of different implementations yielded an accuracy rate of over 99%. However, there are several things to consider when making a final decision on a wearable gadget. The following is a list of features that would make a smart wearable more practical:

- Noninvasive: the gadget should not penetrate or pierce the skin to collect data;
- Compact: the wearable device should not be bulky or large, as its main purpose is to monitor health symptoms without interfering with one’s life activities;
- Affordable: the affordability of the device plays a role in how well it fits into everyday life;
- Robust: the device should be durable enough to handle cold, hot, humid, or dry weather, as well as harsh operating conditions such as light scratches or bumps;
- Ease of use: if the hardware used requires little human input, it should have an intuitive interface;
- Durable power source: the portable device must be powered reliably enough to collect meaningful data over an extended period of time.

On the other hand, the electrocardiogram (ECG) is considered the most effective indicator of cardiovascular disease due to its high accuracy in recording the presence of such disease and its practicality and reliability in detecting it. Conventional ECG signal acquisition relies on electrodes, which can be uncomfortable to wear during normal daily activities. Smart watches and wristbands, on the other hand, are quite effective at capturing ECG signals and are also convenient for a number of other reasons. They are available to everyone and are the best option as they combine a variety of useful features with accurate monitoring of heart rate and other vital signs. Commercially available smart watches and wristbands are cheap and have simple user interfaces. They are small, are not in the way, and do not limit people’s options. In addition, they are equipped with reliable power sources that allow them to last for a long time. Finally, their ability to record a wide range of biometric data makes them an excellent, if not ideal, option for ECG capture devices and thus for predicting CVD parameters.

### 5.2. Latest Tech-Trends and Wearables in CVDs

Alternatively, it is interesting to examine whether or not smart wearables used to control CVD are consistent with current machine learning practices. Several subfields of machine learning were identified as current research areas, but “Explainable AI”, “Federated Machine Learning”, and “Multimodal Machine Learning” were most frequently mentioned. The compliance of smart wearables used to detect CVDs to those topics is discussed in the following sections.

#### 5.2.1. Explainable AI

The more complex AI becomes, the more difficult it becomes for humans to understand and reconstruct the thought process of the algorithm. The entire computational process becomes a so-called “black box”, something that cannot be understood by humans. These black box models are created from scratch using nothing but the raw data. They are so complicated that not even the engineers or data scientists who create them can explain how their artificial intelligence algorithms arrive at their conclusions. Insight into the reasoning behind an AI system’s results can be very helpful. Being able to explain a decision can be critical in allowing stakeholders to challenge or change the conclusion, in meeting regulatory criteria, or in ensuring that the system works as intended by its creators [137,138,139].

In this context, and to address the challenges posed by the black-box nature of AI, ML, and DL models, explainable AI (XAI) is proposed as a viable solution. The goal of XAI is to make the results and outputs generated by machine learning algorithms understandable and reliable to human users. The term refers to a method for describing an AI model along with its intended effects and possible biases. In AI-driven decision-making, it helps describe the precision, fairness, transparency, and outcomes of the model. When it comes to bringing AI models into production, a company’s ability to explain the rationale behind its decisions is critical to building trust with employees and customers. Companies may take a more ethical approach to AI development if AI can be explained [137,138,139].

However, it was found that not a single study mentioned above implemented the explainable AI. While the aforementioned studies were able to achieve high accuracy in diagnosing cardiovascular disease, it may be difficult to implement such wearable technologies into the healthcare cycle if people do not know how the models arrive at such results. In other words, if the results are not explained, the medical community and patients will not have confidence in them, or at least be wary of adopting them.

#### 5.2.2. Federated Machine Learning

The importance of protecting sensitive information has been studied for some time, leading to the development of a variety of protocols for encrypting communications between participants. Differential privacy [140], k-order anonymity [141], homomorphic encryption [142], and other approaches have been developed to protect data before they are transmitted. While several attacks have been uncovered in ML, such as the model inversion attack [143] and the affiliation attack [144], none of them are foolproof, as they can infer raw data by accessing the model.

Federated machine learning, often referred to as federated learning (FL), is a novel idea recently introduced by Google in the machine learning field [145]. The main concept behind FL is to eliminate the exchange of user data between peripherals. FL is a collaborative, distributed/decentralized ML privacy-preserving technology that eliminates the need to transfer data from peripherals to a central server in order to train a model. Instead, the models are sent to the peripheral nodes, where they are trained on the local data, and then sent back to the central aggregation node, where the global model is created without the nodes ever seeing the embedded data. Fortunately, federated learning has emerged as a powerful response to user privacy concerns, paving the way for the collection of additional data to train ML models to improve their accuracy and efficiency.

**Figure 8:**
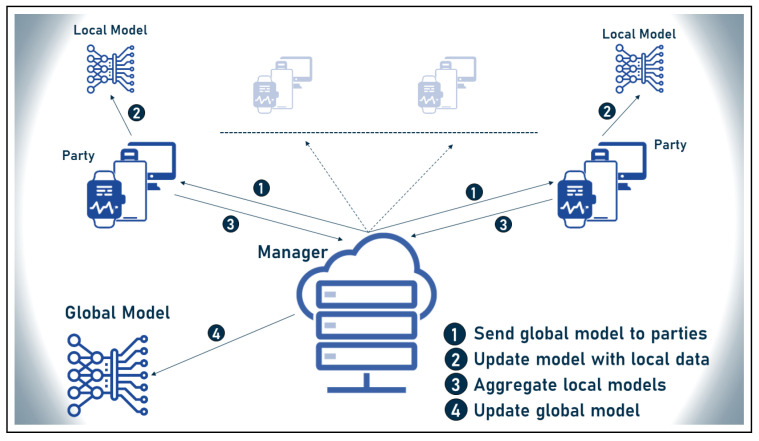
Federated machine learning classical structure.

Furthermore, FL enables training models using data from multiple locations that have data with different structure and composition, also known as data islands, and integrating the information into a global trained model, improving the efficiency of the models. In addition, FL enabled “Learning Transfer”, where models can share their knowledge without having to transfer users’ private data, and made it possible to deal with heterogeneous data scattered in multiple data spaces containing different attributes. The main concept of federated learning is explained in Figure 8 below.

Federated machine learning has shown promising results in the healthcare industry, as indicated in [146,147]. However, none of the studies included in this review addressed the integration of federated learning into wearable devices to make accurate predictions of cardiovascular disease while maintaining privacy. There could be a few reasons for this. For example, FL is still in its infancy and is still vulnerable to various challenges [148,149]. As a result, these factors may slow down the widespread use of FL in smart wearables in cardiology. However, integrating federated learning into smart wearables may lead to the following outcomes:

- Preserving users’ private data, especially health-related data;
- Enabling analysis of data from multiple sources in addition to the vital signs captured by the wearables, such as the patient’s medical history derived from electronic health records and the ECG recorded in real time, to provide more accurate results;
- Building users’ confidence in smart wearables for cardiovascular disease management and subsequent product adoption.

#### 5.2.3. Multimodal Machine Learning

Multimodal machine learning is concerned with integrating different and divergent data sources to benefit from complementary information in a single computational framework that takes care of a single task, and follows this rule in the context of machine learning (ML), a branch of AI. When it comes to predictive capability, the ability to explore many datasets simultaneously leads to more trustworthy and accurate results, making multimodal machine learning an area of high efficiency and amazing potential. To determine a single goal, multimodal machine learning combines information from many modalities [150].

In this context, data fusion is the process of combining information from many databases. “The process of merging data to improve state estimates and projections” [151] is a more precise definition of data fusion. The Joint Directors of Laboratories (JDL) Data Fusion Subpanel concludes that the method of “data fusion” is essential for dealing with many types of data. This description is supported by the authors in [152], who state that any process that deals with linking, correlating, or combining data retrieved from one or more sources to generate improved information is considered a process that employs data fusion. Because the literature on data fusion is still relatively young, there is no general agreement on the optimal way to merge disparate datasets. This is especially true considering that there are four different methods for performing this [151,152]:

- Early fusion: disparate data sources are merged into a single feature vector before being used by a single machine learning algorithm.
- Intermediate fusion: takes place in the intermediate phase between input and output of a ML architecture, when all data sources have the same representation format.
- Late fusion: defines the aggregation of decisions from multiple ML algorithms, each trained with different data sources.
- Hybrid fusion: defines the use of more than one fusion discipline in a single deep algorithm.

**Figure 9:**
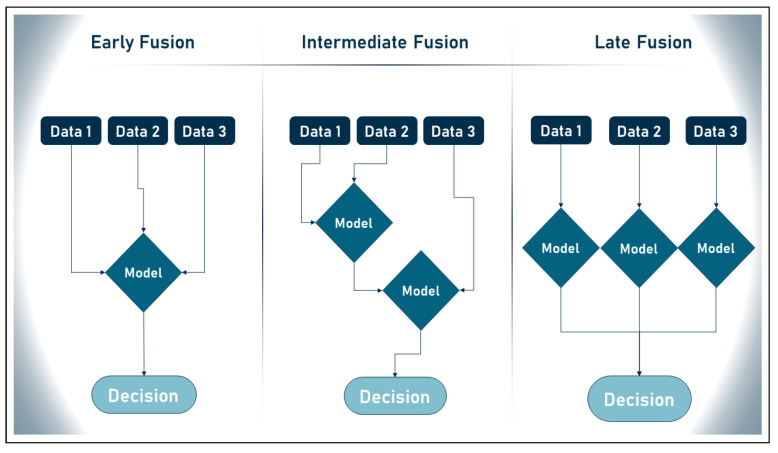
Data fusion different approaches.

The approaches to data fusion defined above are illustrated in Figure 9 below. In addition, none of the smart wearable CVD detection studies reviewed here explored the use of multimodal ML in their algorithms. However, by using this technology, researchers can evaluate many datasets simultaneously, which greatly improves the accuracy of their results. Multimodal ML allows researchers to analyze medical imaging data such as MRIs, ECGs, and EHR data, giving the public more confidence in the accuracy of our AI models.

## 6. Challenges and Future Perspectives

Despite the significant role that smart wearables play in the detection of cardiovascular disease, several issues may arise with their use. In addition, the introduction of new artificial intelligence tools and concepts presents many new opportunities to improve the management of heart disease. In this section, challenges and future prospects are discussed to help future studies select starting points for future investigations.

### 6.1. Challenges

The following are the most common challenges faced by smart wearables in detecting cardiovascular disease. These challenges were identified by analyzing the studies listed in Table 2 and reviewing the literature on smart wearables. Additional information can be obtained from a variety of sources, including, but not limited to [153,154,155,156,157].

#### 6.1.1. Data Privacy and Confidentiality

AI models built into smart wearable technologies work only as well as the information they have access to. While the technical structure of the models themselves—including the cleanliness and suitability of the data—can affect how much data can be used to train AI models, it is generally accepted that more data can lead to more accurate models. In practice, however, there are several obstacles that make data collection the most difficult part of developing AI models. First and foremost is privacy and confidentiality. The security and privacy of personal data are not only strengthened by people, but also by society in general, governments, and companies. Numerous laws and regulations have been enacted to protect personal data, including the European Union’s General Data Protection Regulation (GDPR) [158], the Chinese People’s Republic of China’s Cybersecurity Law [159], the People’s Republic of China’s General Principles of Civil Law [160], Singapore’s PDPA [161], and hundreds of other principles around the world. Although these regulations help protect private information, they pose new challenges to the traditional AI data processing model to varying degrees by making it more difficult to collect data to train models, which in turn makes it more difficult to improve the accuracy of model performance.

#### 6.1.2. Noise and Artifacts

The noninvasive nature of vital signs collection by smart wearables leaves the recordings open to a greater amount of background noise, known as “artifacts”. Artifacts are unwanted signals or signal distributions that distort the actual signal and contribute to the noise in the data, degrading the quality of the data and reducing the performance and accuracy of the smart models. Artifacts can be divided into two categories, depending on where they originate: intrinsic artifacts, which come from the monitored body itself, and extrinsic artifacts, which are caused by the monitored person’s external environment. The origin of artifacts can be divided into many categories [162,163]:

- Intrinsic artifacts (also known as physiological or internal artifacts):
  – Ocular artifacts: created by ocular motions including blinking, horizontal and vertical eye movement, fluttering of the eyes, etc.;
  – Muscle artifacts: caused by things such as sneezing, swallowing, clenching, talking, lifting the eyebrows, chewing, contracting the scalp, etc.;
  – Respiratory artifacts: resulting from an electrode’s movement while breathing, which might manifest as slow, repetitive EEG activity;
  – Sweat artifacts: result of sweat’s electrolyte concentration shifts on the electrode’s surface after contact with the scalp and are obtained in wearables that collect vital signs that are related to skin.
- Extrinsic artifacts (also known as extra-physiological/external artifacts):
  – Motion artifacts: EEG monitoring systems are susceptible to motion artifacts due to the subject’s physical movement;
  – Environmental artifacts: these include, but are not limited to, loss of electrode-to-scalp contact, electrode rupture, electromagnetic wave interference from nearby electrical or electronic equipment, etc.

#### 6.1.3. Data Diversity and Heterogeneity

Research in the field of medicine has shown that the use of multiple vital signs may be more helpful in detecting a disease than the use of a single vital sign. Therefore, combining multiple vital signs in the analysis process could allow for more accurate prediction of cardiovascular disease. Combining ECG signals with medical history data from the electronic health record (EHR) and medical images such as magnetic resonance imaging (MRI) is a robust example of multiple vital signs that can be analyzed together to predict cardiovascular disease. However, these data differ in their nature and structure, or even in the devices used to acquire them. More specifically, ECG data are usually stored in the form of real numbers, while EHR data may be in the form of clinical reports, health tests, or other forms, and MRI images are usually stored in different image formats. In this context, classical machine learning models such as support vector machines are usually well suited for linear data, but it is well known that images can be analyzed with deep learning algorithms such as convolutional neural networks. Therefore, it is a difficult task to analyze these data together given their different formats and structures, even if it is more practical for disease detection.

#### 6.1.4. User Technology Adoption and Engagement

One of the major barriers to the use of smart wearables to detect and predict cardiovascular disease is user acceptance, adoption, and participation. Wearing such sensors is received differently by users due to concerns about privacy, discomfort, ethics, and other contextual factors.

**Figure 10:**
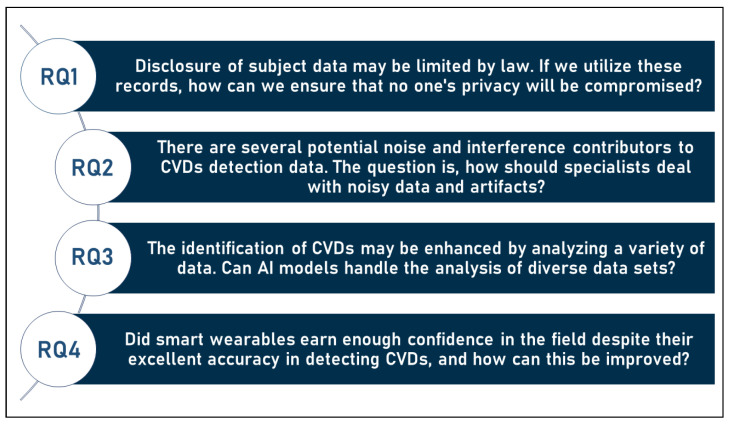
Research questions arising from analysing usage of wearables in CVDs detection.

Therefore, we may characterize the difficulties as the following set of study questions. In addition, those questions are illustrated in Figure 10 below (the symbol RQ in the list below and in Figure 10 refers to the term “research question”):

- **RQ1**: Disclosure of subject data may be limited by law. If we utilize these records, how can we ensure that no one’s privacy will be compromised?
- **RQ2**: There are several potential noise and interference contributors to CVDs detection data. The question is, how should specialists deal with noisy data and artifacts?
- **RQ3**: The identification of CVDs may be enhanced by analyzing a variety of data. Can AI models handle the analysis of diverse datasets?
- **RQ4**: Did smart wearables earn enough confidence in the field despite their excellent accuracy in detecting CVDs, and how can this be improved?

### 6.2. Future Perspectives

Cardiovascular disease detection through smart wearables is now a reality. With the global incidence of this disease and the deaths associated with it, there is a growing need to improve the overall process and take more measures for proactive and preventive methods. More research and development on smart wearables is needed to keep up with the increasing demand.

#### 6.2.1. Preserving Data Privacy and Confidentiality

Newer machine learning methods offer new opportunities to protect the privacy and security of user data. One potential technique that can help solve privacy problems is federated learning (FL). Federated learning, a type of collaborative decentralized machine learning that protects user privacy, does not require data to be transported from edge devices to a central server [149,164,165,166,167]. It is expected that using FL to identify CVDs will make it easier to collect more data, which in turn will improve detection accuracy.

#### 6.2.2. Artifacts Removal and Data Readiness

Before proceeding with signal processing, it is important to eliminate or reduce all artifacts, both extrinsic and intrinsic, that might interfere with the signals. References [163,168,169,170] detail some of the existing implementations that perform this function. In order to clean and preprocess the data to improve the accuracy of cardiovascular disease detection, it is necessary to investigate the automation of noise reduction.

#### 6.2.3. Analysis of Heterogeneous and Diverse Data

Multimodal machine learning is a good solution that allows analyzing data with alternative structures and formats. Since current cardiovascular disease detection and prediction implementations usually analyze only one type of data structure (linear, images, etc.), multimodal machine learning allows analyzing multiple types of data simultaneously to improve the overall result of the intelligent model. Learning a complex task by analyzing data from multiple sources and using complementary knowledge are examples of what multimodal machine learning is capable of. In this context, multimodal datasets are described as information with different structures and formats that come from a variety of sources, each of which contributes a unique set of information (or “modality”) to the overall dataset. Therefore, using the concept of multimodal ML to analyze different data such as ECG, EHR recordings, and MRI images can help increase the accuracy of CVD detection and prediction.

#### 6.2.4. Raising Trust by Enhancing Accuracy, Privacy, and Explainability

Given the prevalence and devastating impact of cardiovascular disease, there is a growing need for practical and viable solutions that can help detect and even predict the onset of these conditions. Consequently, smart wearables have proven to be viable in this area, providing both continuous and real-time monitoring without interfering with daily life routines. However, there is a great need to improve the prediction of CVDs using smart wearables, whether through increased accuracy, better explainability, or by addressing other issues that hinder their adoption by users, such as privacy and ethical constraints. This is a well-known fact that does not need further explanation, because when it comes to health, users are only willing to use tools that are highly accurate, understandable, private, and reliable. In other words, greater trust and wider use of smart wearables as tools for predicting CVDs will result from improved accuracy, reliability, feasibility, privacy, and explainability of such devices.

**Figure 11:**
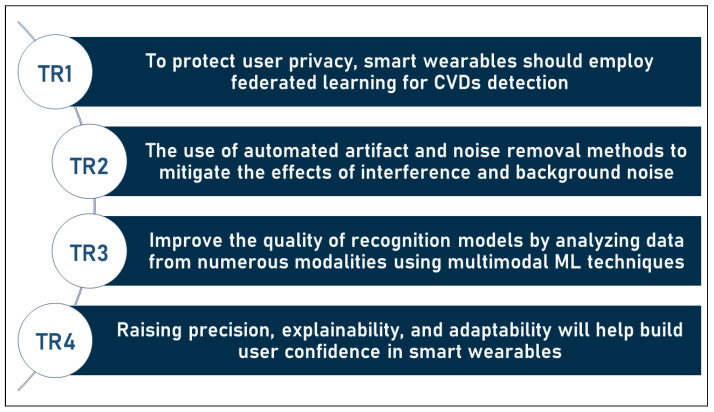
Research topics that may serve as solutions to the challenges in the domain.

For this reason, we may summarize the outlook into the following trending research topics. In addition, those research topics are illustrated in Figure 11 below (the symbol TR in the list below and in Figure 11 refers to the term “trending research topic”):

- **TR1:** To protect user privacy, smart wearables should employ federated learning for CVDs detection;
- **TR2:** The use of automated artifact and noise removal methods to mitigate the effects of interference and background noise;
- **TR3:** Improve the quality of recognition models by analyzing data from numerous modalities and sources using multimodal ML techniques;
- **TR4:** Raising precision, explainability, and adaptability will help build users’ confidence in smart wearables.

**Figure 12:**
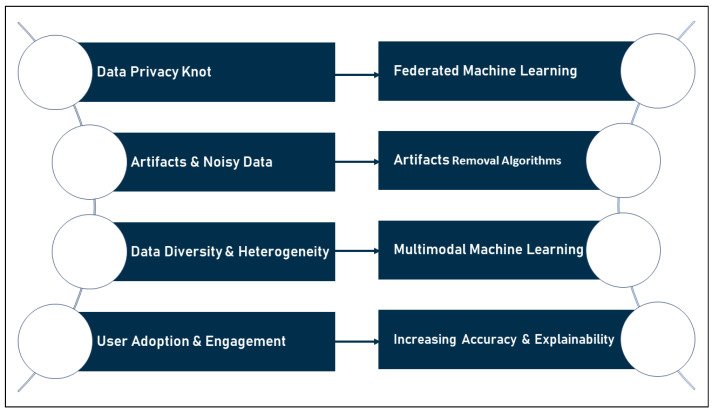
Challenges–future solutions chart.

Figure 12 below summarizes the challenges–future solutions relationship and illustrates how future views may act as potential solutions in the domain, all of which can assist to enhance research into the use of smart wearables in the detection of CVDs.

## 7. Conclusions

Recently, the use of smart wearables in the diagnosis and prediction of cardiovascular disease has received increasing attention. This is partly due to the technological potential of smart wearables and partly due to the data processing power of artificial intelligence and its derivatives, machine learning and deep learning. In this research, we thoroughly investigated the use of smart wearables to treat fatal heart diseases. The review of the research area showed the high practicality and effectiveness of such methods, reflecting the growing interest that has surged in recent years. However, given the challenges and limitations discussed in this review, there is a large window for improvement that smart wearables should undergo to prove their feasibility and reliability. Increasing accuracy, automating noise reduction, solving privacy issues, dealing with heterogeneity, and improving explainability are interesting topics that should be considered when trying to promote the use of smart wearables in the management of CVDs. As a result, this review provides a brief overview of a number of relevant topics that can be used as recommendations for further research.

## Figures and Tables

**Table 1:** Success reasons and success points of smart wearables.

	Powered By	Capabilities
Smart wearables	Low power consumption Compact size Adaptable styles Robustness	Continuous functionality Long-term Monitoring Real-time data sensing Communication with Internet

**Table 2:** Implementations of smart wearables in detection of CVDs.

Ref#	Year	Disease(s) Targeted	Vital Signs Collected	Hardware Employed	Smart Model(s) Used	Training Dataset(s)	Results Metrics
[39]	2010	Atrial Fibrillation	Electrocardiogram	A wearable vest including dry foam ECG acquisition device A mobile phone (Nokia N85)	Not Identified	PhysioNet MIT-BIH dataset	Sensitivity: 94.56% Positive Predictive Value: 99.22%
[40]	2010	Right Bundle Branch Block Beats Premature Ventricular Contraction Paced Beats Fusion of Paced and Normal Beats	Electrocardiogram	Plug-In-Based GUI Platform: An Alive Bluetooth ECG heart monitor and Amoi E72 Microsoft Windows Mobile 5 Smartphone Machine-Learning-Based Platform: An Alive Bluetooth ECG heart monitor and an HTC Microsoft Windows Mobile 6 Smartphone	Multilayer Perceptron	PhysioNet MIT-BIH dataset	Accuracy > 90%
[41]	2010	Sinus Tachycardia Sinus Bradycardia Cardiac Asystole Atrial Fibrillation Wide QRS Complex	Electrocardiogram	A three-lead ECG device that contain two main parts: NCTU ECG Aquisition tool as the data acquisition (DAQ) unit and a wireless-transmission unit. Medi-Trace 200, Kendall are also used to read the ECG from the body	Not Identified	Dataset collected at MUSE ECG system (GE health care, USA) in China Medical University (CMUH) database	Accuracy > 92%
[42]	2011	Premature Ventricular Contraction Atrial Premature Contraction	Electrocardiogram Electroencephalogram Respiratory Rate Skin Temperature	Wearable Sensor Node and it consists of seven modules: analog front-end circuits for four physiological signals, a radio communication module, a storage module, and MSP430F2618 as microcontroller unit (MCU) Smartphone: HTC HD2 with a 1 GHz CPU and 448 MB RAM (can be replaced with any android, Windows or IOS phone)	Hidden Markov Model Layered Hidden Markov Model	PhysioNet MIT-BIH dataset	Sensitivity: 99.72% Positive Predictive Value: 99.64%
[43]	2011	Congestive Heart Failure Malignant Ventricular Ectopy Ventricular Tachycardia	Electrocardiogram	A wireless ECG sensor S3C6400 mobile phone HBE-ZigbeX motes as a wireless sensor network	Multilayer Perceptron	PhysioNet MIT-BIH dataset	BIDMC Congestive Heart Failure: 100% Malignant Ventricular Ectopy: 90.9% Ventricular Tachyarrhythmia: 83.3%
[44]	2015	Atrial Fibrillation	Electrocardiogram	Rejiva ECG wearable sensor and a smartphone	Support Vector Machines	PhysioNet MIT-BIH dataset	Specificity: 77.25% Sensitivity: 93.13%
[45]	2016	Atrial Fibrillation	Electrocardiogram Photoplethysmogram	Samsung Simband wrist band smart watch	Elastic Net Logistic model	Private Data	Accuracy: 95% Sensitivity: 97% Specificity: 94% AUROC: 99%
[46]	2016	Myocardial Ischemia	Electrocardiogram	A smart cloth composed of four units: Smart cloth unit to measure physiological signal-ECG signal Signal control unit to control and memorize the status of the device by an ultra-low power MCU and SD card to save the signal data Signal sensing unit that has a motion tracking sensor module to capture the accelerometer signal Wireless connection unit to transmit the data A smartphone	Neural Network	PhysioNet MIT-BIH dataset PhysioNet MIT-BIH Normal Sinus Rhythm dataset	Accuracy > 76%
[47]	2017	Atrial Fibrillation	Electrocardiogram Photoplethysmogram	Samsung Simband wrist band smart watch	Convolutional Neural Network Elastic Net Logistic model	Private Data	Accuracy: 91.8%
[48]	2017	Heart Attack	Electrocardiogram Body Temperature	Device composed of pulse sensor, a temperature sensor, an Arduino, and a Low Energy (LE) Bluetooth A smartphone	Not Identified	Private Data	
[49]	2017	Ventricular Premature Complex Atrial Premature Complex Ventricular Fibrillation Atrial Fibrillation	Electrocardiogram	Bio Clothing One, XYZ life BC1	Artificial Neural Networks	PhysioNet American Heart Association database PhysioNet Creighton University Ventricular Tachyarrhythmia database PhysioNet MIT-BIH dataset PhysioNet MIT-BIH Noise Stress Test database	Accuracy > 75%
[50]	2017	Atrial Fibrillation	Electrocardiogram	Wrist bracelet designed for the purpose: based on the ultra low power series Microcontroller STM32L471RG	Support Vector Machines	Private Data	Accuracy: 95%
[51]	2017	Atrial Fibrillation	Audio Signal in Radial Artery	The PAG monitoring device consists of four components audiogram sensor: Panasonic capacitive microphone analog-digital converter: Embedded in Atmega328P microprocessor: Atmega328P chip data storage unit A smartphone	Convolutional Neural Network	Dataset collected at National Cheng Kung University Hospital (NCKUH), Tainan, Taiwan.	Accuracy: 98.92%
[52]	2018	Myocardial Infarction	Electrocardiogram	ECG sensor using AD8232 and Espressif ESP-32 Wi-Fi + BLE module	Convolutional Neural Network	PhysioNet PTB Diagnostic ECG Database	Accuracy: 84%
[53]	2018	Ventricular Arrhythmia Junctional Arrhythmia Supraventricular Arrhythmia Arrhythmias	Electrocardiogram	a smart clothing consisting of cloth carrier, biosen sor platform, and smart terminals. In biosensor platform, ADI ECG analog front-end (ADAS1001) is used for obtaining the ECG signals, Microcontroller (STM32) is used to realize the data processing and a Bluetooth module is available for data transfer	Deep Neural Network with a Softmax Regression model	PhysioNet MIT-BIH dataset	Accuracy > 94%
[54]	2018	Hypertension	Heart Rate	A waist belt comprised of three kinds of sensors: three dry electrodes, a 3-axis accelerometer and two pressure sensors with different sensitivities	Logistic Regression Support Vector Machines	Private Data	Accuracy: 93.33%
[55]	2018	Atrial Fibrillation	Electrocardiogram Photoplethysmogram	Samsung gear device wearable device	Convolution–Recurrent Hybrid Model (CRNN)	Private Data	Accuracy > 98%
[56]	2018	Atrial Fibrillation	Electrocardiogram	A smart shirt equipped with ECG sensors A smartphone		Dataset collected at the Dongsan Medical Center in South Korea	Accuracy: 98.2%
[57]	2018	Ventricular Tachycardia Ventricular Bradycardia Premature Atrial Contractions Premature Ventricular Contractions	Electrocardiogram	for ECG Sensing: ECG body sensor with analog conditioning circuit (AD8232), Microcontroller unit (MCU) (PIC12F1822), Bluetooth module (HC-06), and charging controller module for analysis and display: processing and displaying unit of that process the ECG signal and display it on thin film transistor (TFT) liquid crystal display (LCD) consisting of Rpi computer, Bluetooth module, TFT screen, and power supply	Support Vector Machines	PhysioNet MIT-BIH dataset	Accuracy: 96.2%
[58]	2019	Myocardial Infarction Heart Failure Arrhythmias Fusion Beats Supraventricular Ectopic Beats Ventricular Ectopic Beats	Electrocardiogram Heart Rate Respiratory Rate	A patch with electronic circuit is built for the purpose and proposed in the article and an Android smartphone and a cloud server for data storage and further analysis	Convolutional Neural Network	PhysioNet PTB Diagnostic ECG Database St Petersburg INCART 12-lead Arrhythmia Database	Accuracy: 98.7%
[59]	2019	Atrial Fibrillation	Electrocardiogram	A patch with electronic circuit is built for the purpose and proposed in the article and an Android smartphone and a cloud server for data storage and further analysis	Decision Tree	PhysioNet MIT-BIH dataset	Accuracy > 97.18%
[60]	2019	Atrial Fibrillation Atrial Flutter Ventricular Fibrillation	Electrocardiogram	A wearable ECG sensing device and an Android smartphone and a cloud server for data storage and further analysis	Convolutional Neural Network	PhysioNet MIT-BIH dataset	Accuracy > 94%
[61]	2019	Atrial Fibrillation	Electrocardiogram	Smart vest equipped with two ECG sensing units	Long Short-Term Memory	PhysioNet dataset of the 2017 Computing in Cardiology Challenge	Sensitivity: 83.82% Specificity: 97.84% F1-score: 81.43%
[62]	2019	Supraventricular Ectopic Beats Ventricular Ectopic Beats	Electrocardiogram	ECG sensing device with a smartphone or tablet	Long Short-Term Memory	PhysioNet MIT-BIH dataset	Accuracy > 79%
[63]	2019	Atrial Fibrillation	Heart Rate	Commercial HR Sensor	Long Short-Term Memory	PhysioNet Atrial Fibrillation Database (AFDB)	Accuracy: 98.51%
[64]	2019	Arrhythmias Congestive Heart Failure	Electrocardiogram	One lead ECG sensor	Convolutional Neural Network	PhysioNet MIT-BIH dataset PhysioNet MIT-BIH Normal Sinus Rhythm database	Accuracy: 93.75%
[65]	2019	Arrhythmias	Electrocardiogram	A device composed of a single-lead heart rate monitor front end AD8232 chip, Atmel’s ATmega128 as a microcontroller and a BLE module A smartphone is also used	Support Vector Machines K-Nearest Neighbors Logistic Regression Random Forest Decision Tree Gradient Boosting Decision Tree	PhysioNet MIT-BIH dataset	Accuracy > 77%
[66]	2019	Atrial Fibrillation	Photoplethysmogram	Wearable wristband device	Support Vector Machines	Private Data	Accuracy: 90%
[67]	2020	Atrial Bigeminy Atrial Fibrillation Atrial Flutter Ventricular Bigeminy Heart Block Ventricular Trigeminy Ventricular Flutter Ventricular Tachycardia Supraventricular Tachyarrhythmia Idioventricular Rhythm Paced Beats Nodal (A-V Junctional) Rhythm	Electrocardiogram	SparkFun Single Lead Heart Rate Monitor AD8232 as the data acquisition device Smartphone as a gateway to the server	Convolutional Neural Network	PhysioNet MIT-BIH dataset	Accuracy: 94:13%
[68]	2020	Atrial Fibrillation	Electrocardiogram Photoplethysmogram	Amazfit Healthband 1S for ECG and PPG sensing smartphone for data reception and analysis	Convolutional Neural Network	Dataset collected at Peking University First Hospital	Sensitivity: 80.00% Specificity: 96.81% Accuracy: 90.52%
[69]	2020	Left Bundle Branch Block Beats Right Bundle Branch Block Beats Atrial Premature Contraction Ventricular Premature Contraction Paced Beats Ventricular Escape Beats	Electrocardiogram	A sensing device composed from a single lead heart rate monitor AD8232 and interfaced with NodeMCU development board having ESP8266 microcontroller capable of connecting to internet via WiFi Smartphone for the analysis of the data	Convolutional Neural Network	PhysioNet MIT-BIH dataset	Accuracy > 90%
[70]	2020	Cardiovascular Risk	Electrocardiogram Electroencephalogram Electromyogram Heart Rate Blood Pressure Respiratory Rate Blood Sugar Level Oxygen Saturation Level Cholesterol Levels	Wearable medical sensors and a wearable smart watch	Convolutional Neural Network	UCI Cleveland Heart Diseases Dataset	Accuracy: 98.5%
[71]	2020	Atrial Fibrillation	Electrocardiogram Photoplethysmogram Photoplethysmogram Oxygen saturation Level Body Temperature	The sensing device used is composed of three parts: AD8232r for ECG detection, ADS1115 analog-to-digital converter and SX1276 LoRa chip that transmits the data to the fog device The fog device: a low-cost raspberry pi system integrated with Intel Neural Compute Stick 2 (NCS 2) that is capable of handling deep learning algorithms	Convolutional Neural Network	PhysioNet dataset of the 2017 Computing in Cardiology Challenge	Accuracy: 90%
[72]	2020	Cardiovascular Risk	Electrocardiogram Blood Pressure	An ECG sensing device built with AD8232 unit A smart watch raspberry pi with SX1272 unit to transmit the data for LoRa gateway	Convolutional Neural Network	UCI Cleveland Heart Diseases Dataset	Accuracy: 98.2%
[73]	2020	Aortic Stenosis Mitral Insufficiency Mitral Stenosis Tricuspid Regurgitation	Electrocardiogram Photoplethysmogram Gyrocardiography Seismocardiogram	Shimmer 3 from Shimmer Sensing for ECG detection A three-axis MEMS accelerometer: (Kionix KXRB5-2042, Kionix, Inc.) to measure the SCG signal A three-axis MEMS gyroscope (Invensense MPU9150, Invensense, Inc.) to record the GCG signal An ear-lobe photoplethysmography (PPG) sensor	Decision Tree Random Forest Neural Network	Dataset collected at Columbia University Medical Center (CUMC)	Accuracy > 90%
[74]	2020	Left Bundle Branch Block Beats Right Bundle Branch Block Beats Atrial Escape Beats Nodal (Junctional) Escape Beats Atrial Premature Beats Aberrated Atrial Premature Beats Nodal Premature Beats Supraventricular Premature Beats Premature Ventricular Contractions Ventricular Escape Beats Fusion of Ventricular and Normal Beats Paced Beats Fusion of Paced and Normal Beats	Electrocardiogram	A sensing device composed of AD8232 single-lead three-electrode ECG Heart Rate monitor and a ESP8266 Wi-Fi module used to provide wireless data transmission access to the Arduino Nano and is used to connect it to the cloud	Convolutional Neural Network	PhysioNet MIT-BIH dataset	Accuracy: 99.625% Sensitivity: 97.736% Specificity: 99.713% Precision: 97.835%
[75]	2020	Ventricular Ectopic Beats Arrhythmias	Electrocardiogram	Sensing device composed of Raspberry Pi for processing, ADS1115 as Analog to Digital Converter and AD8232 as ECG sensor	Convolutional Neural Network	PhysioNet MIT-BIH dataset	Accuracy: 95.76%
[76]	2020	Premature Atrial Contractions Premature Ventricular Contractions Atrial Fibrillation	Electrocardiogram Photoplethysmogram	7-lead Holter monitor (Rozinn RZ153+ Series, Rozinn Electronics Inc., Glendale, NY, USA) Smartwatch (Simband 2, Samsung Digital Health, San Jose, CA, USA)	Random Forest Support Vector Machines	Dataset collected at the ambulatory cardiovascular clinic at the University of Massachusetts Medical Center (UMMC)	Best Model Accuracy: 94%
[77]	2020	Arrhythmias	Electrocardiogram	Sensing device built using Raspberry Pi 3 model B+ and two ECG sensors AD8232 with a pulse sensor and an analog digital converter ADS1015	Support Vector Machines Naïve Bayes Artificial Neural Networks	PhysioNet MIT-BIH dataset	Best Model Accuracy: 97.8%
[78]	2020	Atrial Fibrillation	Electrocardiogram	the wearable system is composed to work on a prototype developed by Medicaltech srl (Rovereto, Italy)	A Custom model based on Thresholding of Shannon Entropy values	PhysioNet MIT-BIH dataset	Sensitivity: 99.2% Specificity: 97.3%
[79]	2020	Atrial Fibrillation	Electrocardiogram	The sensing device is composed of Raspberry pi 3, Arduino UNO, AD8232 single lead ECG sensor, HC-05 Bluetooth, biomedical sensor pad and battery	Long Short-Term Memory	PhysioNet MIT-BIH dataset	Accuracy: 97.57%
[80]	2020	Atrial Escape Beats Junctional Escape Beats Left Bundle Branch Block Beats Right Bundle Branch Block Beats Atrial Premature Beats Aberrated Atrial Premature Beats Junctional Premature Beats Supraventricular Premature Beats Premature Ventricular Contractions Ventricular Escape Beats Fusion of Ventricular and Normal Beats Paced Beats Fusion of Paced and Normal Beats	Electrocardiogram	Moto 360 NanoPi Neo Plus2 Raspberry Pi Zero	Long Short-Term Memory	PhysioNet MIT-BIH dataset	Accuracy > 98.6 %
[81]	2020	Supraventricular Arrhythmia Atrial Fibrillation Arrhythmias	Electrocardiogram	A wearable sensing device composed of AD8232 as an ECG sensor, MCP3008 ias an ADC and Raspberry Pi as a computing unit	Support Vector Machines	UCI Cleveland Heart Diseases Dataset	Accuracy: 72.41%
[82]	2020	Arrhythmias	Electrocardiogram Body Temperature Heart Rate Blood Oxygen Level	A sensing device composed of: Temperature sensor: MLX90614 Heart rate and blood oxygen sensors: MAX30100 ECG sensor: AD8232 Inter-Integrated Circuit (I2C) communication protocol Microcontroller: Arduino UNO Wireless transmission: Wi-Fi chip ESP8266 A smartphone	Long Short-Term Memory Convolutional Neural Network	PhysioNet MIT-BIH dataset	Accuracy: 99.05%
[83]	2020	Premature Ventricular Contraction	Electrocardiogram	A wireless 3-lead ECG sensor from Shimmer Sensing	Support Vector Machines	PhysioNet MIT-BIH dataset	Sensitivity: 96.51% Predictive Value: 81.92%
[84]	2020	Atrial Fibrillation Syncope	Electrocardiogram	A sensing device composed of: The SparkFun AD8232 ECG sensing unit Arduino Mega 2560 microcontroller Raspberry Pi 3 board ADXL345 triple-axis accelerometer HC-05 Bluetooth sensor A smartphone	Long Short-Term Memory	PhysioNet MIT-BIH dataset	Accuracy: 97.61%
[85]	2021	Atrial Fibrillation	Pulse Plethysmogram	Wrist-type pulse wave monitor (type: Smart TCM-I, product by: Shanghai Asia & Pacific Computer Information System CO, Ltd, Shanghai, China)	Time Synchronous Averaging	Private Data	Accuracy: 98.4%
[86]	2021	Cardiovascular Risk	Photoplethysmogram	Pulse rate sensor with ATmega32 microcontroller	Support Vector Machines Naïve Bayes Random Forest Decision Tree Logistic Regression Artificial Neural Networks Recurrent Neural Networks	Dataset collected at Framingham University	Accuracy: 94.9%
[87]	2021	Ventricular Ectopic Beats Supraventricular Ectopic Beats	Electrocardiogram	Ternary second-order delta modulator circuits	Support Vector Machines	PhysioNet MIT-BIH dataset	Accuracy > 98%
[88]	2021	Premature Atrial Contractions Premature Ventricular Contractions Atrial Fibrillation Ventricular Tachycardia Sinus Bradycardia Atrial Tachycardia	Electrocardiogram	A custom-built ECG Signal acquisition circuit	Gramian Angular Fields (GAFs) Deep Residual Network (ResNet)	PhysioNet MIT-BIH dataset LTAF database Simulated Data (Prosim2 Vital Sign Simulator)	Accuracy: 98.1% Sensitivity: 97.6% Specificity: 99.7% F1 Score: 97.6%
[89]	2021	Arrhythmias Congestive Heart Failure	Electrocardiogram	ARDUINO UNO ECG SENSOR AD8232 DISPOSABLE ECG ELECTRODES	Support Vector Machines	PhysioNet dataset of the 2016 Computing in Cardiology Challenge	Accuracy: 98%
[90]	2021	Atrial Fibrillation	Electrocardiogram	A consumer-grade, single-lead heart belt (Suunto Movesense, Suunto, Vantaa, Finland)	Not Identified	Private Data	Accuracy 97.8%
[91]	2021	Atrial Fibrillation Atrial Flutter Supraventricular Tachycardia Ventricular Tachycardia	Electrocardiogram	ECG247 Smart Heart Sensor	Not Identified	Private Data	Accuracy > 95%
[92]	2021	Heart Attack	Electrocardiogram Heart Rate Body Temperature Blood Pressure	A device composed of ECG, heart rate, body temperature, and blood pressure sensors	Not Identified	Private Data	Accuracy: 83%
[93]	2021	Atrial Fibrillation Ventricular Bradycardia Ventricular Tachycardia Bundle Branch Block	Electrocardiogram	HealthyPiV3 biosensors	Convolutional Neural Network	PhysioNet MIT-BIH dataset PhysioNet PAF Prediction Challenge Database for AF records PhysioNet PTB Diagnostic ECG Database PhysioNet dataset of the of 2015 bradycardia Challenge PhysioNet Fantasia Database and PAF Prediction Challenge Database for healthy signals	Accuracy > 98.75%
[94]	2021	Heart Attack	Electrocardiogram	AD8232 ECG sensor	Sequential Covering Algorithm	PhysioNet PTB-XL dataset	F1 Score: 87.8%
[95]	2021	Heart Attack	Electrocardiogram Body Temperature Activity Parameters Oxygen Saturation Level	Composed of different sensors to collect different vital signs which are: LM35, MPU 6050, MAX30100 and AD8232 respectively	Support Vector Machines Linear Regression K-Nearest Neighbors Naïve Bayes	Private Data	Accuracy: 80%
[96]	2021	Ventricular Premature Beats Supraventricular Premature Beats Atrial Fibrillation	Electrocardiogram	IREALCARE2.0 Wearable ECG Sensor	Time-Span Convolutional Neural Network Recurrent Neural Networks	Private Data	F1 Score: 86.5% Precision: 87.7% Recall: 86.8%
[97]	2021	Cardiovascular Risk	Electrocardiogram Oxygen Saturation Level	Composed of AD8232 (ECG sensor) and MAX30102 (SPO2 sensor)	Convolutional Neural Network Convolutional Neural Network	PhysioNet MIT-BIH dataset	Shallow CNN Accuracy: 96.06% Deep CNN Accuracy: 98.47%
[98]	2021	Heart Failure Hypertension Atrial Fibrillation Peripheral Artery Disease Myocardial Contraction	Heart Rate Activity Parameters	GENEActiv and Activinsights Band (Activinsights Ltd., Kimbolton, UK)	Not Identified	To be collected	To be provided
[99]	2021	Atrial Fibrillation	Heart Rate Respiratory Rate	BioHarness 3.0 by Zephyr	Support Vector Machines	PhysioNet MIT-BIH dataset	Sensitivity: 78% Specificity: 66%
[100]	2021	Atrial Fibrillation Bigeminy Arrhythmias	Electrocardiogram	AD8232	Decision Tree	Private Data	Accuracy > 95%
[101]	2021	Atrial Fibrillation Atrial flutter Left Bundle Branch Block Beats Wolff-Parkinson-White Syndrome Atrial Premature Contraction Premature Ventricular Contraction	Electrocardiogram	A smart vest equipped with AD8232 ECG Sensor	Shallow Wavelet Scattering Network (ScatNet)	PhysioNet MIT-BIH dataset	Accuracy > 96%
[102]	2021	Tachycardia	Heart Rate Respiratory Rate Blood Oxygen Level	Medical-grade wearable embedded system (SensEcho, Beijing SensEcho Science & Technology Co Ltd)	Long Short-Term Memory	Medical Information Mart for Intensive Care III (MIMIC-III)	Up to 80% accuracy 2 h before onset of Tachycardia
[103]	2021	Atrial Fibrillation	Photoplethysmogram	Samsung Galaxy Active 2 Watch	Convolutional Neural Network	Private Data	Accuracy 91.6% Specificity 93.0% Sensitivity 90.8%
[104]	2021	Arrhythmias	Electrocardiogram	A chest sticker that is composed from BMD101 ECG sensing device with YJ33 power supply, BQ24072 as a power source and JDY-30 as a Bluetooth module	Convolutional Neural Network	PhysioNet MIT-BIH dataset	Accuracy: 99.83%
[105]	2022	Supraventricular Ectopic Beats Ventricular Ectopic Beats Fusion Beats	Electrocardiogram	Polar H10	Decision Tree Gradient Boosting k-Nearest Neighbors Multilayer Perceptron Random Forest Support Vector Machines	PhysioNet MIT-BIH dataset	Best Model Accuracy: 99.67%
[106]	2022	Supraventricular Ectopic Beats Ventricular Ectopic Beats Fusion Beats	Electrocardiogram	Polar H10	Decision Tree Gradient Boosting k-Nearest Neighbors Multilayer Perceptron Random Forest Support Vector Machines	PhysioNet MIT-BIH dataset	Best Model Accuracy: 99%
[107]	2022	Heart Failure Reduced Ejection Fraction	Electrocardiogram	Galaxy Watch Active & AppleWatch 6	Convolutional Neural Network	Private Data	Area Under Curve 93.4%
[108]	2022	Atrial Fibrillation	Photoplethysmogram Electrocardiogram	Samsung GalaxyWatch Active 2 Chest ECG Patch	Hybrid Decision Model	Private Data	Average: 67.8%
[109]	2022	Atrial Fibrillation	Photoplethysmogram	Custom-built device that contains the PPG sensor MAX30102	Convolutional Neural Network	Data obtained from Kaunas University of Technology	F1-score: 94%
[110]	2022	Atrial Fibrillation	Electrocardiogram	Firstbeat Bodyguard 2, Firstbeat Technologies	Not Identified	Private Data	Accuracy 98.7% Sensitivity 99.6%, Specificity 98.0%
[111]	2022	Supraventricular Ectopic Beats Ventricular Ectopic Beats	Electrocardiogram	Custom-built device that contains the ECG AFE sensor	Artificial Neural Networks Decision Tree K-Nearest Neighbors	PhysioNet MIT-BIH dataset	Accuracy: 98.7%
[112]	2022	Atrial Fibrillation	Photoplethysmogram	Apple Watch	Gradient Boosting Decision Tree	Private Data	Accuracy: 94.16%
[113]	2022	Congestive Heart Failure Atrial Fibrillation	Electrocardiogram	AD8232 sensor	Random Forest	PhysioNet MIT-BIH dataset	Accuracy: 85%
[114]	2022	Cardiovascular Risk	Photoplethysmogram Body Temperature Activity Parameters	Custom-built device with Pulse Sensor, DS18B20 temperature sensor and ADXL 1335 as accelerometer sensor	Naïve Bayes Decision Tree K-Nearest Neighbors Support Vector Machines	Kaggle Human Gait Dataset Kaggle Heart Disease Prediction Dataset	Accuracy: 82%
[115]	2022	Cardiovascular Risk	Heart Rate Respiratory Rate Blood Oxygen Level	Not identified (WBAN)	Enhanced version of Recurrent Neural Network named ERNN	Private Data	Accuracy: 96%
[116]	2022	Cardiovascular Risk	Electrocardiogram Electroencephalogram Body Temperature Blood Oxygen Level Respiratory Rate Blood Sugar Level	A custom-built device equipped with electrocardiogram sensor, electroencephalogram sensor, an electro-mammography sensor, an oxygen level sensor, a temperature sensor, a respiration rate sensor, and a glucose level sensor	Long Short-Term Memory	UCI Cardiac Arrhythmia Dataset	Average Positive Predictive Value: 96.77% Average Negative Predictive Value: 95.12% Average Sensitivity: 95.30%
[117]	2022	ST Elevation Myocardial Infarction (STEMI)	Electrocardiogram Motion Data	Custom-built device with 3-axis accelerometer (ADXL355), 3-axis gyroscope (LSM6DS3) and single-lead ECG sensors	Logistic Regression	Private Data	Sensitivity: 73.9% Specificity: 85.7%
[118]	2022	Cardiovascular Risk	Electrocardiogram Motion Data	A custom-built device with accelerometers, Galvanic Skin Response (GSR) and electrocardiograms (ECG) sensors	Mixed Kernel Based Extreme Learning Machine (MKELM)	Private Data	Accuracy: 99.5%
[119]	2022	Cardiovascular Risk	Heart Rate	Wrist Strap & Rohm BH1790GLC-EVK-001 Development board BH1790GLC Optical heart rate sensor	Convolutional Neural Network	Simulated Data	F1-Score: Up to 99%
[120]	2022	Myocardial Infarction Dilated Cardiomyopathy Hypertension	Pulse Plethysmogram	PTN-104 PPG sensor	Support Vector Machines K-Nearest Neighbors Decision Tree	Private Data	Accuracy: 98.4% Sensitivity: 96.7% Specificity: 99.6%
[121]	2022	Cardiovascular Risk	Heart Rate Blood Sugar Level	Heart rate sensor by Sunrom Electronics Glucose monitor by Medtonic	Naïve Bayes K-Nearest Neighbors Support Vector Machines Random Forest Artificial Neural Networks	Private Data	Accuracy: 97.32% Recall: 97.58% Precision: 97.16% F1-Measure: 97.37% Specificity: 96.87% G-Mean: 97.22%
[122]	2022	Cardiovascular Risk	Electrocardiogram	A custom-built device composed of ECG sensor (AD8232) and other components	Random Forest	UCI Cleveland Heart Diseases Dataset	Accuracy: 88%
[123]	2022	Cardiovascular Risk	Heart Rate Oxygen Saturation Level Systolic Pressure Diastolic Pressure	Custom-built soft transducer equipped with MAX30100 SpO2 and HR monitor sensor	Long Short-Term Memory	Kaggle dataset (Not Specified)	Accuracy > 93%
[124]	2022	Cardiovascular Risk	Electrocardiogram Blood Pressure Pulse Plethysmogram Body Temperature	Custom-built device equipped with ECG sensor, TMP117 temperature sensor, Honeywell’s 26 PC SMT blood pressure sensor, and a pulse oximeter	Recurrent Neural Networks	UCI Cleveland Heart Diseases Dataset	Accuracy: 99.15% Precision: 98.06% Recall: 98.95% Specificity: 96.32% F1-Score: 99.02%
[125]	2022	Congenital Heart Disease	Electrocardiogram Seismocardiogram	Custom-built chest wearable sensor equipped with ECG sensor (ADS1291; Texas Instruments, Dallas, TX) and seismocardiogram sensor (ADXL355; Analog Devices, Norwood, MA)	Ridge Regression	Private Data	-

**Table 3:** Vital signs used in studies with count and percentages.

Vital Sign	Count	Percentage
Electrocardiogram	69	79.31%
Photoplethysmogram	15	17.24%
Heart rate	13	14.94%
Body temperature	8	9.20%
Respiratory rate	7	8.05%
Oxygen saturation level	5	5.75%
Blood oxygen level	4	4.60%
Blood pressure	4	4.60%
Activity parameters	3	3.45%
Blood sugar level	3	3.45%
Electroencephalogram	3	3.45%
Pulse plethysmogram	3	3.45%
Motion data	2	2.30%
Seismocardiogram	2	2.30%
Audio signal in radial artery	1	1.15%
Cholesterol levels	1	1.15%
Diastolic pressure	1	1.15%
Electromyogram	1	1.15%
Gyrocardiography	1	1.15%
Skin temperature	1	1.15%
Systolic pressure	1	1.15%

**Table 4:** Smart Models Used in Studies.

Smart Model	Count
Convolutional neural network	23
Support vector machines	20
Decision tree	10
Long short-term memory	10
Random forest	9
K-nearest neighbors	8
Artificial neural networks	5
Naïve Bayes	5
Not identified	5
Logistic regression	4
Multilayer perceptron	4
Recurrent neural networks	3
Elastic net logistic model	2
Gradient boosting	2
Gradient boosting decision tree	2
Neural network	2
A custom model based on thresholding of Shannon entropy	1
Convolution–recurrent hybrid model (CRNN)	1
Deep neural network with a softmax regression model	1
Deep residual network (ResNet)	1
Enhanced version of recurrent neural network named ERNN	1
Gramian angular fields (GAFs)	1
Hidden Markov model	1
Hybrid decision model	1
Layered hidden Markov model	1
Linear regression	1
Mixed-kernel-based extreme learning machine (MKELM)	1
Ridge regression	1
Sequential covering algorithm	1
Shallow wavelet scattering network (ScatNet)	1
Time-synchronous averaging	1
Time-span convolutional neural network	1

## Data Availability

The study did not report any data.
